# Neonatal outcome in vaginal breech labor at 32 + 0—36 + 0 weeks of gestation: a nationwide, population-based record linkage study

**DOI:** 10.1186/s12884-022-04547-9

**Published:** 2022-03-16

**Authors:** Anna Toijonen, Seppo Heinonen, Mika Gissler, Georg Macharey

**Affiliations:** 1 University of Helsinki, Helsinki, Finland; 2 Department of Obstetrics and Gynecology, University Hospital (HUS), University of Helsinki, Haartmaninkatu 2, 00290 Helsinki, Finland; 3 National Institute for Health and Welfare (THL), Helsinki, Finland

**Keywords:** Preterm delivery, Preterm labor, Breech presentation, Vaginal labor, Adverse outcome

## Abstract

**Background:**

In many countries, vaginal breech labor at term is an option in selected cases. However, the safety of vaginal breech labor in preterm is still unclear. Therefore our study aimed to evaluate the safety of vaginal breech labor in late preterm deliveries.

**Design:**

A retrospective register-based study.

**Setting:**

Maternity hospitals in Finland, 2004–2017.

**Participants:**

The study population included 762 preterm breech deliveries at 32 + 0—36 + 6 gestational weeks according to the mode of delivery, 535 (70.2%) of them were born vaginally in breech presentation, and 227 (29.8%) were delivered by non-urgent cesarean section.

**Methods:**

The study compared short-term neonatal adverse outcomes of singleton vaginal breech deliveries with non-urgent cesarean deliveries at 32 + 0 to 36 + 6 weeks of gestation. An odd ratio with 95% confidence intervals was calculated to estimate the relative risk of adverse outcomes.

**Outcome measures:**

Neonatal death, an arterial umbilical pH below seven, a five-minute Apgar score below four and seven, admission to neonatal intensive care unit, neonatal intubation, neonatal antibiotic therapy, neonatal birth trauma, respiratory distress syndrome, neonatal convulsions, cerebral ischemia, hypoxic-ischemic encephalopathy, congenital hypotonia, and a composite of severe adverse outcomes.

**Results:**

A five-minute Apgar scores below seven were increased in vaginal breech labor at 32 + 0 to 36 + 6 weeks of gestation compared to non-urgent cesarean sections (aOR 2.48, 95% CI 1.08–5.59). Neonatal antibiotic therapy, the admission to neonatal intensive care unit, and neonatal respiratory distress syndrome were decreased after vaginal breech labor compared to the outcomes of non-urgent cesarean section (neonatal antibiotic therapy aOR 0.60, 95% CI 0.40–0.89; neonatal NICU admission aOR 0.47, 95% CI 0.33–0.68; respiratory distress syndrome aOR 0.30, 95% CI 0.19–0.48).

**Conclusion:**

Vaginal breech labor at 32 + 0—36 + 6 gestational weeks does not increase severe neonatal short-term morbidity or mortality compared to cesarean section.

## Introduction

In developed countries, 5–9% of all deliveries occur preterm [1]. Preterm pregnancy itself is also known as the most significant risk factor for breech presentation at delivery [2–5]. Risk factors for breech presentation in preterm deliveries are premature rupture of membranes, preeclampsia, oligohydramnios, fetal growth restriction, and congenital anomalies [6]. Many of these factors are associated with adverse perinatal outcomes in vaginal preterm breech delivery [7].

In term pregnancies, fetal breech presentation at delivery is a risk factor for adverse neonatal outcomes [8, 9] as vaginal breech labor is associated with increased neonatal short-term morbidity compared to cephalic deliveries [2, 10]. In the Term Breech Trial 2000, cesarean section was recommended for breech deliveries at term to reduce the risks of adverse outcomes of vaginal breech labor [11]. Since then the results of the Term Breech Trial have been disputed [12] and nowadays, in well-selected cases, vaginal breech labor at term is an option in many countries [13–15]. In singleton preterm breech deliveries without other complications, the optimal mode of birth is still widely debated. A lower Apgar score and an increased need for admission to the neonatal intensive care unit have been associated with preterm vaginal breech labor compared to the neonates born in cephalic presentation [16]. However, a trial of vaginal moderate to late preterm breech labor has not increased neonatal adverse outcomes compared to a planned cesarean section [16]. Nevertheless, other studies have reported reduced neonatal deaths with a cesarean section for preterm breech fetuses [17–20]. Furthermore, a recently published European cohort study (2019) implied that in very preterm breech labor, fetuses born by cesarean section instead of vaginally had reduced mortality and morbidity [21].

Most of the existing studies compromise deliveries in very preterm gestational weeks. The other problem of the previous studies is the small patient numbers. Our research aims to reduce the uncertainty regarding preterm breech deliveries at 32 + 0—36 + 6 weeks of gestation and investigate if vaginal preterm labor at 32 + 0—36 + 6 weeks of gestation is feasible.

## Methods

Our study is a retrospective register-based study with 762 singleton preterm deliveries from 32 + 0 to 36 + 6 weeks of gestation. The study was performed using data from the Finnish Institute for Health and Welfare. The Finnish Institute for Health and Welfare maintains the national medical birth register and the hospital discharge register. Reporting clinical data on national registers is compulsory for all maternity hospitals. All maternal, obstetric, and neonatal data on inpatient and outpatient care in public hospitals is reported in the hospital discharge register. As national data protection law requires, The Finnish Institute for Health and Welfare authorized using the data. The study methods complied with all the relevant guidelines and regulations. The need of informed consent was waived by the regional research committee of the medical faculty of Helsinki University because registered persons were not contacted. The ethical approval of the study was accepted by the National Institute for Health and Welfare and the regional research committee of the medical faculty of Helsinki University. The data that support the findings of this study are available from the Finnish Institute for Health and Welfare but restrictions apply to the availability of these data, which were used under license for the current study, and so are not publicly available. Data are however available from the authors upon reasonable request and with the authorization of from the Finnish Institute for Health and Welfare.

We reviewed all singleton breech deliveries born vaginally or by non-urgent cesarean section at 32 + 0 to 36 + 6 weeks of gestation over a study period of thirteen years from 2004 to 2017 in Finland. We limited the study population to singleton deliveries, and we excluded deliveries with a fetus in other presentations than breech. We excluded the emergency cesarean sections to ensure that cesarean sections performed due to urgent clinical indications which were not associated with breech presentation itself would not affect the results. In addition, we excluded all deliveries complicated with placental abruption (ICD-10 O71.0, O71.1) and all fetal congenital anomalies (as defined in the Register of Congenital Malformations), as these complications might have affected neonates surviving and measured adverse outcomes.

The neonates born vaginally in breech presentation were compared to the neonates born by non-urgent cesarean section in breech presentation. The primary outcome was short-term neonatal outcome defined as neonatal death within 27 days of birth, an arterial umbilical pH below seven, a five-minute Apgar score below seven and below four, the admission to neonatal intensive care unit (NICU), neonatal intubation, neonatal antibiotic therapy, and a composite of severe adverse outcomes (an arterial umbilical pH below 7, a five-minute Apgar score below 4, and neonatal death within 27 days). Furthermore, neonatal birth trauma (ICD-10 P10-15), respiratory distress syndrome (RDS, ICD-10 P22), neonatal convulsions (ICD-10 P90), cerebral ischemia (ICD-10 P91.0), hypoxic-ischemic encephalopathy (HIE, ICD-10 P91.6), congenital hypotonia (ICD-10 P94.2), and one or more of the diagnoses of intraventricular hemorrhage (IVH, ICD-10 P10.2), RDS, neonatal convulsions, cerebral ischemia, and HIE were included in primary outcomes. The criteria of neonatal intubation are standardized in Finnish hospitals. In addition, the following maternal determinants were analyzed in our study: maternal age, parity, pre-pregnancy body mass index (BMI), smoking, diabetes mellitus type one or two (ICD-10 E10, E11), gestational diabetes (ICD-10 O24.4), preeclampsia or pregnancy-induced high blood pressure (ICD-10 O13, O14), history of induced abortion or miscarriage, assisted reproduction therapy, and history of cesarean section. The following fetal and obstetric variables were also analyzed: oligohydramnios (ICD-10 O41.0), fetal sex, birth weight below 10^th^ percentile of standard deviation, birth weight above 90^th^ percentile, preterm premature rupture of membranes (PPROM) (ICD-10 O42), administration of antenatal corticosteroids, and epidural analgesia.

SPSS for Windows V0.19.0, Chicago, Illinois, United States of America was used to calculate the statistical analyses. Statistical differences in variables were calculated with a chi-Squared test or Fisher’s exact test as appropriate. Odds ratios (ORs) with a corresponding 95% confidence interval (CIs) were calculated, and *p*-values ≤ 0.05 were defined as statistically significant.

## Results

Our study included 762 breech deliveries at 32 + 0—36 + 6 weeks of gestation, 535 (70.2%) of them had vaginal breech labor, and 227 (29.8%) were delivered by non-urgent cesarean section.

The mothers who had vaginal breech labor had less likely a history of cesarean section (OR 0.18, 95% CI 0.11–0.29), assisted reproduction therapy (OR 0.45, 95% CI 0.23–0.89), or diagnosed with diabetes mellitus type one (OR 0.02, 95% CI 0.01–0.10) than the women in the non-urgent cesarean section group. Furthermore, the pregnancies which had vaginal breech labor had fewer cases complicated with oligohydramnios (OR 0.14, 95% CI 0.05–0.40) and preeclampsia / pregnancy-induced high blood pressure (OR 0.09, 95% CI 0.05–0.17) than the pregnancies delivered by non-urgent cesarean section. PPROM preceded vaginal breech labor more often than it did in the non-urgent cesarean section group (OR 1.58, 95% CI 1.06–2.35). The neonates born vaginally in breech presentation were less likely to have birth weight below 10^th^ or above 90^th^ percentile than the neonates in the non-urgent cesarean section group (birth weight < 10^th^ percentile OR 0.19, 95% CI 0.11–0.32); birth weight > 90^th^ percentile OR 0.19, 95% CI 0.07–0.50). The mothers who had vaginal breech labor at 32 +—36 + 6 weeks of gestation had less administered antenatal corticosteroids to accelerate the maturation of the fetus` lungs than the mothers who were performed a non-urgent cesarean section (OR 0.22, 95% CI 0.16–0.32). Additional information on maternal, fetal, and obstetric characteristics of the studied and control group are shown in Table 1.

**Table 1:** Maternal, fetal, and obstetric characteristics of vaginal breech deliveries and non-urgent cesarean sections at 32 + 0—36 + 6 gestational weeks 2004–2017 in Finland

	**Vaginal** **breech labor** **n / %** **535 / 100%**	**Non-urgent cesarean** **section** **n / %** **227 / 100%**	***P*-value**	**Crude OR**	**95%** **confidence** **interval**
**Maternal age < 25y**	8 / 1.5%	2 / 0.9%	0,712	1.71	0.36—8.11
**Maternal age ≥ 35y**	116 / 21.7%	53 / 23.3%	0,712	0.91	0.63- 1.32
**Nulliparous**	287 / 57.7%	132 / 58.1%	0,253	0.83	0.61—1.14
**Multipara ≥ 3 deliveries**	41 / 7.7%	17 / 7.5%	0,934	1.03	0.57—1.85
**Maternal smoking**	76 / 14.2%	36 / 15.9%	0,556	0.88	0.57—1.35
**Maternal BMI ≥ 30**	25 / 4.7%	15 / 6.6%	0,273	0.69	0.36—1.34
**History of induced abortion**	69 / 12.9%	22 / 9.7%	0,212	1.38	0.83—2.29
**History of miscarriage**	114 / 21.3%	60 / 26.4%	0,123	0.75	0.53—1.08
**History of cesarean section**	30 / 5.6%	56 / 24.7%	< 0,001	0.18	0.11—0.29
**Assisted reproduction technology**	19 / 3.6%	17 / 7.5%	0,019	0.45	0.23—0.89
**Diabetes mellitus type I**	2 / 0.4%	32 / 14.1%	< 0,001	0.02	0.01- 0.10
**Diabetes mellitus type II**	2 / 0.4%	1 / 0.4%	0,893	0.85	0.08—9.40
**Gestational diabetes mellitus**	53 / 9.9%	15 / 6.6%	0,144	1.55	0.86—2.82
**Oligohydramnios**	5 / 0,9%	14 / 6.2%	< 0,001	0.14	0.05—0.40
**Preeclampsia / pregnancy-induced high blood pressure**	12 / 2.2%	46 / 20.3%	< 0,001	0.09	0.05—0.17
**PPROM**	132 / 24.7%	39 / 17.2%	0,023	1.58	1.06—2.35
**Birth weight < 10th percentile**	22 / 7.6%	42 / 18.5%	< 0,001	0.19	0.11—0.32
**Birth weight > 90th percentile**	6 / 1.1%	13 / 5.7%	< 0,001	0.19	0.07—0.50
**Epidural analgesia**	221 / 41.3%	0 / 0%	< 0,001		
**Administration of antenatal corticosteroids**	88 / 16.4%	106 / 46.7%	< 0,001	0.22	0.16—0.32
**Neonatal female sex**	264 / 49.3%	124 / 54.6%	0,182	0.81	0.59—1.10

*OR* Odds Ratio, *BMI* Body Mass Index, *PPROM* Preterm Premature Rupture Of Membranes

The neonates who were born vaginally in breech presentation at 32 +—36 + 6 weeks of gestation had an increased risk of having five-minute Apgar scores below seven compared to the neonates in the control group (aOR 2.48, 95% CI 1.08–5.59). However, the difference between the groups in five-minute Apgar scores was no longer significant when comparing Apgar scores below four. The neonates born vaginally in breech presentation had a decreased risk of antibiotic treatment (aOR 0.60, 95% CI 0.40–0.89) and the need for admission to NICU (aOR 0.47, 95% CI 0.33–0.68) compared to the neonates born by non-urgent cesarean section. Half (49.5%) of the neonates who were born vaginally in breech presentation needed admission to NICU, whereas the admission to NICU was 71.4% in the non-urgent cesarean section group. There was one neonatal death in the group that had vaginal breech labor but no deaths in the non-urgent cesarean section group. No significant difference in the need for neonatal intubation, umbilical pH, or a composite of severe adverse outcomes was discovered between the groups. Neonatal birth trauma was diagnosed in eight neonates who were born vaginally in breech presentation at 32 +—36 + 6 weeks of gestation (vaginal breech labor 1.5% vs. non-urgent cesarean Sect. 0%). However, the difference in neonatal birth trauma between the groups was not statistically significant. The neonates in vaginal breech group had less diagnoses of neonatal respiratory distress syndrome than neonates in the non-urgent cesarean section group (aOR 0.30, 95% CI 0.19–0.48). The incidence of neonatal convulsions, cerebral ischemia, HIE, or congenital hypotonia did not differ significantly between the groups. The short-term adverse outcomes of breech deliveries at 32 + 0—36 + 6 gestational weeks are presented in Table 2.

**Table 2:** The short-term adverse outcomes of vaginal breech deliveries and non-urgent cesarean sections at 32 + 0—36 + 6 gestational weeks 2004–2017 in Finland

	**Vaginal** **breech labor** **n / %** **535 / 100%**	**Non-urgent cesarean** **section** **n / %** **227 / 100%**	***P*-value**	**Adjusted OR**^**a**^ **(95% confidence interval)**
**Neonatal death 0–27 days**	1 / 0.2%	0 / 0.0%	0,515	
**Arterial umbilical** **pH < 7**	6 / 1.1%	0 / 0.0%	0,109	
**5 min Apgar < 4**	7 / 1.3%	1 / 0.4%	0,282	2.77 (0.30—25.58)
**5 min Apgar < 7**	41 / 7.7%	8 / 3.5%	0,033	2.48 (1.08—5.59)
**Intubation**	15 / 2.8%	5 / 2.2%	0,635	1.24 (0.38—6.06)
**Antibiotics newborn**	111 / 20.7%	68 / 30.0%	0,006	0.60 (0.40—0.89)
**Neonatal NICU admission**	265 / 49.5%	162 / 71.4%	< 0,001	0.47 (0.33—0.68)
**Severe adverse outcome** ** (arterial umbilical pH < 7, 5 min Apgar < 4, neonatal death 0-27d)**	14 / 2.6%	1 / 0.4%	0,048	7.11 (0.83—60.79)
**Birth trauma**	8 / 1.5%	0 / 0.0%	0,064	
**RDS**	63 / 11.8%	62 / 27.3%	< 0,001	0.30 (0.19—0.48)
**Neonatal convulsions**	1 / 0.2%	0 / 0.0%	0,515	
**Cerebral ischemia**	0 / 0%	0 / 0%		
**HIE**	0 / 0%	0 / 0%		
**Congenital hypotonia**	0 / 0%	0 / 0%		
**Any dg of IVH, RDS, neonatal convulsions, cerebral ischemia, or HIE**	63 / 11.8%	62 / 27.3%	< 0,001	0.30 (0.19—0.48)

*OR* Odds Ratio, *NICU* Neonatal Intensive Care Unit, *RDS* Respiratory Distress Syndrome, *HIE* Hypoxic-Ischemic Encephalopathy, *dg* diagnose, *IVH* Intraventricular Hemorrhage due to birth injury

^a^Adjusted for maternal age, parity, BMI, smoking, diabetes, oligohydramnios, child’s sex, and birth weight < 10th percentile

## Discussion

Our study’s most important finding is that vaginal breech labor at 32 + 0—36 + 6 weeks of gestation does not seem to increase severe neonatal short-term morbidity or mortality compared to the outcomes of non-urgent cesarean section. Overall, the need for treatment in a neonatal intensive care unit is high due to fetal prematurity, but neonatal deaths at 32 +—36 + 6 weeks of gestation are rare.

Our study showed an increase in the five-minute Apgar score below seven in the neonates who were born vaginally in breech presentation at 32 + 0—36 + 6 weeks of gestation compared to the neonates born by non-urgent cesarean section. However, there was no longer a significant difference in the five-minute Apgar scores when the limit was reduced to four or below. Another Finnish research in 2018 indicated similar results in breech deliveries at 32 + 0 to 36 + 6 weeks of gestation; the five-minute Apgar scores were comparable between neonates born after a trial of vaginal breech delivery and cesarean section [16]. However, Toivonen and colleagues found that one-minute Apgar scores were lower in breech neonates born vaginally than the vertex controls. In addition, our results showed no difference in the arterial umbilical pH between the groups, which indicates that acidosis was not increased after vaginal breech labor. This is in contrary to the venous umbilical pH and base excess results of Vidovics and colleagues (2014) who compared outcomes of preterm breech neonates delivered vaginally or by cesarean section at 24–37 gestational weeks [22]. However, low patient numbers in their study did not enable further statistical analysis. Besides in our study, women who were permitted to give birth vaginally with a fetus in breech presentation are most likely low-risk patients without acknowledged obstetric risks and this might reflect to the results.

The neonates in the vaginal breech delivery group did not differ in neonatal death (0–27 days) or a composite of severe adverse outcomes from the neonates born by non-urgent cesarean section. Likewise, Kayem and colleagues (2015) found no higher risk for neonatal mortality or severe morbidity after vaginal labor in preterm breech fetuses [23]. On the contrary, a systematic review of 2014 indicated that the cesarean section in preterm breech fetuses decreases neonatal mortality compared to vaginal labor [17]. Also, Thanh and colleagues (2019) found cesarean section a protective factor for perinatal death in preterm breech deliveries [18]. However, these three studies also included deliveries below 30 gestational weeks, and lower gestational weeks might have affected the contrary results.

Vaginal breech labor at 32 + 0—36 + 6 weeks of gestation did not increase asphyxia-related morbidity, such as IVH, HIE, or cerebral ischemia, compared to non-urgent cesarean sections. On the contrary, neonatal RDS was diagnosed less in neonates in the vaginal breech group than in neonates who were born by non-urgent cesarean section. Birth injuries were more common in neonates born vaginally in breech presentation than in neonates in the cesarean section group. However, the difference was not significant and the absolute risk of birth trauma was small. A meta-analysis 2016 found vaginal breech labor at term to associate with neonatal birth trauma and asphyxia as they compared planned vaginal breech deliveries to planned cesarean sections [24]. However, the heterogeneity of the studies included in the meta-analysis might have affected the results. Furthermore, the management of breech labor might vary in different countries. Another study 2020 consisting of thirty-four preterm deliveries found brachial plexus birth injury to associate with breech presentation at labor [25]. Still the patient number was small, the study included also extremely and very preterm deliveries, and the prognosis of brachial plexus injury was good [25].

Our study indicated that vaginal breech labor at 32 + 0—36 + 6 weeks of gestation decreased the need for neonatal antibiotic treatment or NICU admission comparing to the controls in the cesarean section group. The pregnancies which had vaginal breech labor had less fetal growth restriction, oligohydramnios, preeclampsia, and diabetes mellitus type 1 than the non-urgent cesarean section group. These factors are associated with adverse neonatal short-term outcomes [26–28]. Oligohydramnios is linked to increased fetal distress due to the umbilical cord’s compression and low five-minute Apgar scores in literature [29]. Besides, a low amniotic fluid amount is associated with fetal intrauterine growth restriction (IUGR) [30]. The preterm and term pregnancies complicated with IUGR, despite the presentation, have at least doubled neonatal mortality risk compared with pregnancies without fetal growth restriction [31]. It has been shown that IUGR fetuses in term have a higher likelihood of adverse outcomes in vaginal breech labor than IUGR fetuses born by planned cesarean section [32]. In addition, in vaginal preterm breech labor, fetal growth restriction has been associated with higher mortality [33]. Preeclampsia is a known risk factor for poorer neonatal outcomes [34]. In our results, the incidence of maternal preeclampsia or pregnancy-induced high blood pressure was higher in the cesarean section group. Preeclampsia is associated with abnormal placentation, intrauterine growth restriction, and bronchopulmonary dysplasia of the fetus [35]. Fetuses with such obstetric risk factors might not tolerate the stress of vaginal labor due to placental insufficiency. However, our results might be exposed to selection bias if a cesarean section is recommended for pregnancies with complications such as fetal growth restriction, oligohydramnios, preeclampsia, and diabetes mellitus type one. PPROM occurred more often in the pregnancies which had vaginal breech labor. The mothers whose pregnancies were complicated with PPROM are usually administered antibiotic prophylaxis, and in the case of intrauterine infections, a cesarean section is often recommended [36]. If intrauterine infection is suspected, the neonates are often admitted to NICU and the neonatal antibiotic treatment is started. Furthermore, cesarean section is associated with the risk of neonatal respiratory problems such as neonatal respiratory distress syndrome which aligns with our results [37]. Neonates suffering from early respiratory distress are often admitted to NICU where the need for neonatal antibiotic therapy is easily recognized. These factors altogether might explain the decreased need for antibiotic treatment and the admission to NICU in neonates who were born vaginally in breech presentation compared to those born by non-urgent cesarean section.

Our study included over 750 preterm breech deliveries from thirteen years and offered an excellent sight of the safety of preterm vaginal breech labor. Another advantage is that all the Finnish birth hospitals are public and the treatment equal. However, the retrospective approach exposes our study to the typical bias of such analyses. Also, more patients would have strengthened the results and diminished the possible bias in some cases. Despite having national data from a long period, there is a possibility that we did not have enough power to detect the difference in rare cases, such as mortality. Furthermore, we did not receive the information on the intended birth mode due to the restrictions of the database, and therefore it is possible that few women might have delivered vaginally because labor progressed too rapidly to perform a cesarean section. Another limitation might be that practitioners may have favored a cesarean section for the preterm pregnancies with obstetric complications or underlying maternal reasons. Still, this possible selection bias does not invalidate our study’s main results or the importance of risk evaluation. Our analysis indicates that with individual risk assessment, vaginal breech labor at 32 + 0—36 + 6 gestational weeks can be feasible. However, further studies with long-term outcomes are required.

## Conclusion

Vaginal breech labor at 32 + 0—36 + 6 gestational weeks does not increase severe neonatal short-term morbidity or mortality compared to cesarean section. Therefore, we recommend considering the possibility of vaginal preterm breech labor at 32 + 0—36 + 6 gestational weeks in selected cases without risk factors for adverse outcomes, such as fetal or maternal comorbidity, fetal heart rate abnormalities, or chorioamnionitis.

## Data Availability

The data that support the findings of this study are available from the Finnish Institute for Health and Welfare but restrictions apply to the availability of these data, which were used under license for the current study, and so are not publicly available. Data are however available from the author upon reasonable request (anna.toijonen@helsinki.fi) and with the authorization of from the Finnish Institute for Health and Welfare. More information on the authorization application to researchers who meet the criteria for access to confidential data can be found at Findata, the Health and Social Data Permit Authority: https://www.findata.fi/en/

## Abbreviations

OR: Crude odds ratio
CI: Confidence interval
aOR: Adjusted odds ratio
ICD-10: International Statistical Classification of Diseases and Related Health Problems 10th Revision
NICU: Neonatal intensive care unit
BMI: Body mass index
PPROM: Preterm premature rupture of membranes
IUGR: Intrauterine growth restriction
HIE: Hypoxic-ischemic encephalopathy
IVH: Intraventricular hemorrhage due to birth injury
